# Intensive monitoring of duloxetine: results of a web-based intensive monitoring study

**DOI:** 10.1007/s00228-012-1313-7

**Published:** 2012-06-12

**Authors:** Linda Härmark, Eugène van Puijenbroek, Kees van Grootheest

**Affiliations:** 1 Netherlands Pharmacovigilance Centre Lareb, Goudsbloemvallei 7, 5237 MH ’s-Hertogenbosch, The Netherlands; 2 Department of Pharmacy, Division of Pharmacotherapy and Pharmaceutical Care, University of Groningen, A. Deusinglaan 1, 9713 AV, Groningen, The Netherlands

**Keywords:** Post-marketing surveillance, Intensive monitoring, Web, Duloxetine, Adverse drug reactions

## Abstract

**Purpose:**

Duloxetine (Cymbalta^®^) is a serotonin (5-HT) and norepinephrine (NE) re-uptake inhibitor indicated for the treatment of depression, diabetic peripheral neuropathic pain and general anxiety disorder. The aim of this study is to gain insight in the user and safety profile of duloxetine in daily practice, reported by patients via a web-based intensive monitoring system during their first 6 months of use.

**Methods:**

First-time users of duloxetine were identified through the first dispensing signal in the pharmacy. Patient demographics and information about drug use and adverse drug reactions, ADRs, were collected through electronic questionnaires sent 2 and 6 weeks, 3 and 6 months after the start of duloxetine administration. ADRs were quantified and signal detection was performed on a case by case basis.

**Results:**

Three hundred and ninety-eight patients registered for the study; 69.1 % were female. Depression was the main indication. Three hundred and three patients (76.1 %) filled in at least one questionnaire and 78.9 % of these reported an ADR. Serious ADRs were reported by 4 patients. Three new signals were identified, amenorrhea, shock-like paraesthesias and micturition problems.

**Conclusions:**

Web-based intensive monitoring is an observational prospective cohort study mirroring the use and ADRs of duloxetine in daily practice. This study indicates that duloxetine is a relatively safe drug as used by patients for six months in daily practice, but the aforementioned signals need to be evaluated in more detail.

## Introduction

Duloxetine (Cymbalta^®^) is registered in the European Union for the treatment of major depressive disorder, diabetic peripheral neuropathic pain and generalised anxiety disorder [1]. It is a serotonin (5-HT) and norepinephrine (NE) re-uptake inhibitor with almost equal affinity for binding to NE and 5-HT transport sites, with little affinity for other receptors such as muscarinic, histaminergic, alpha-adrenergic, dopaminergic, serotonergic and opioid receptors, suggesting that it might have a more benign adverse drug reaction profile compared with other antidepressive drugs [2].

The efficacy and safety of duloxetine for the treatment of the registered indications were investigated in clinical trials [1, 3–6]. Clinical trials are primarily designed to prove efficacy. For detection of adverse drug reactions (ADRs), clinical trials have a number of limitations, including a homogeneous population that does not mirror the target population concerning age, gender, comorbidity and comedication, limited sample size and a limited duration [7]. Because of these limitations it is essential to monitor the safety of duloxetine in clinical practice in order to get a clear picture of its ADR profile.

Spontaneous reporting has been the backbone of pharmacovigilance ever since the thalidomide disaster 50 years ago. In a spontaneous reporting system health care professionals and increasingly also patients can submit reports of ADRs. These reports can lead to the detection of a new signal. A signal is defined by the WHO as “Reported information on a possible causal relationship between an adverse event and a drug, the relationship being unknown or incompletely documented” [8].

Spontaneous reporting is a passive form of drug surveillance, where one is dependent on the willingness of health care professionals and patients to report. In order to gain more information about a certain drug, a more active form of drug surveillance is necessary [9, 10].

In 2006, the Netherlands Pharmacovigilance Centre Lareb, which is responsible for the collection and analysis of spontaneous reports in the Netherlands, introduced a web-based intensive monitoring system called Lareb Intensive Monitoring (LIM). LIM is a non-interventional prospective observational cohort that follows users of certain drugs during a certain period of time. In LIM, patients eligible for inclusion are identified in community pharmacies through a first dispensing signal. The patient receives information about the study and if willing to participate, the patient registers online. After registration, questionnaires are sent by e-mail at specific points in time. In these questionnaires questions are asked about patient characteristics, drug use and possible ADRs. The LIM methodology has been described in more detail elsewhere [11, 12]. The aim of this study is to gain insight into the user and safety profile of duloxetine in daily practice, reported by patients via a web-based intensive monitoring system during the first 6 months of use.

## Materials and methods

### Study population

The population consisted of first-time users of duloxetine, identified through the first dispensing signal in intensive monitoring participating pharmacies between 1 November 2006 and 30 April 2008. Data were collected between 1 November 2006 and 31 October 2008.

### Data collection

When registering for the study, patients were asked to provide an e-mail address, which was used for all further correspondence. During registration, patient characteristics and information about duloxetine use and concomitant drug use were collected. After registration, the patient received questionnaires by e-mail 2 and 6 weeks, and 3 and 6 months after starting the drug, where information about possible ADRs due to duloxetine use was collected. If the patient did not fill in the questionnaire immediately, a reminder was sent 5 days later. If a questionnaire was not completed 4 weeks after the reminder, the patient was considered “lost to follow-up” for that questionnaire. If the patient stopped the use of duloxetine, or in the event of death of the patient or if the patient actively chose to stop his participation in the study, the patient did not receive any more questionnaires. The participation in the study was then considered to be completed.

The indication and ADRs were coded using the Medical Dictionary for Regulatory activities (MedDRA) on a Lower Level Term (LLT) level by a qualified assessor [13]. Study drug and co-medication were coded using the Dutch Drug dictionary, Z-index [14]. If a report was judged to be serious according to the Council for International Organisations of Medical Sciences (CIOMS) criteria, which includes (prolongation of) hospitalisation, life-threatening events, events leading to death, disabling events, congenital abnomalities, and other medically significant events [15], and was also assessed as being serious by the assessor, a copy of the report was exported to the national database containing all spontaneous reports, where it was handled according to the regulations regarding serious adverse drug reaction reports. The workflow of Lareb Intensive Monitoring has been described in more depth elsewhere [12].

### Analysis

The frequencies were calculated for gender, age, drug strength used, daily dose and past use of drugs for depression and neuropathic pain. The number of patients reporting a possible ADR, the percentage of serious ADRs, and the incidence of different ADRs were calculated. Even though a patient could report the same adverse drug reactions in all four questionnaires, one specific reaction was only counted once for each individual when calculating incidences. The possible ADRs were divided into labeled or not labeled according to the European Public Assessment Report (EPAR) [1]. Reactions that were not labeled and reactions that were labeled but for other reasons were considered to be of potential interest (selection made by one pharmacist [LH] and one physician [EP]) were analyed on a case by case basis. Labeled reactions were considered to be of interest if frequency differences were found between the cohort and the EPAR.

A comparison between the patients who only filled in the registration form and the patients who provided data on whether or not they had experienced any possible ADRs was made on the basis of age, gender and daily dosage. Significance was declared at the *p* < 0.05 α level. Data were retrieved using Microsoft Access. Statistical analysis was performed using SPSS version 17.

## Results

Between 1 November 2006 and 30 April 2008, 398 patients registered for the duloxetine study; 69.1 % of these were female. The average age was 47.0 (SD 12.3 ) years, ranging from 14 to 82 years. 66.7 % of the patients used duloxetine for depression, 16.1 % for neuropathic pain and 4.3 % for fibromyalgia.

As much as 81.4 % of the population cohort used duloxetine capsule 30 mg and 16.6 % were on 60 mg. In 3.0 % of the cases the capsule strength used was not specified. The average daily dosage was 49.1 mg. Two hundred and thirty-nine patients answered the question asking if they had used any drugs previously for the treatment of depression and/or neuropathic pain. Of the patients who answered the question, 97 (40.6 %) patients had used other drugs for the same indication in the past. The most commonly used drugs were SSRIs, including venlafaxine (55 patients), tri-cyclic antidepressants (13), other anti-depressants (12), and benzodiazepines (10).

**Fig. 1:**
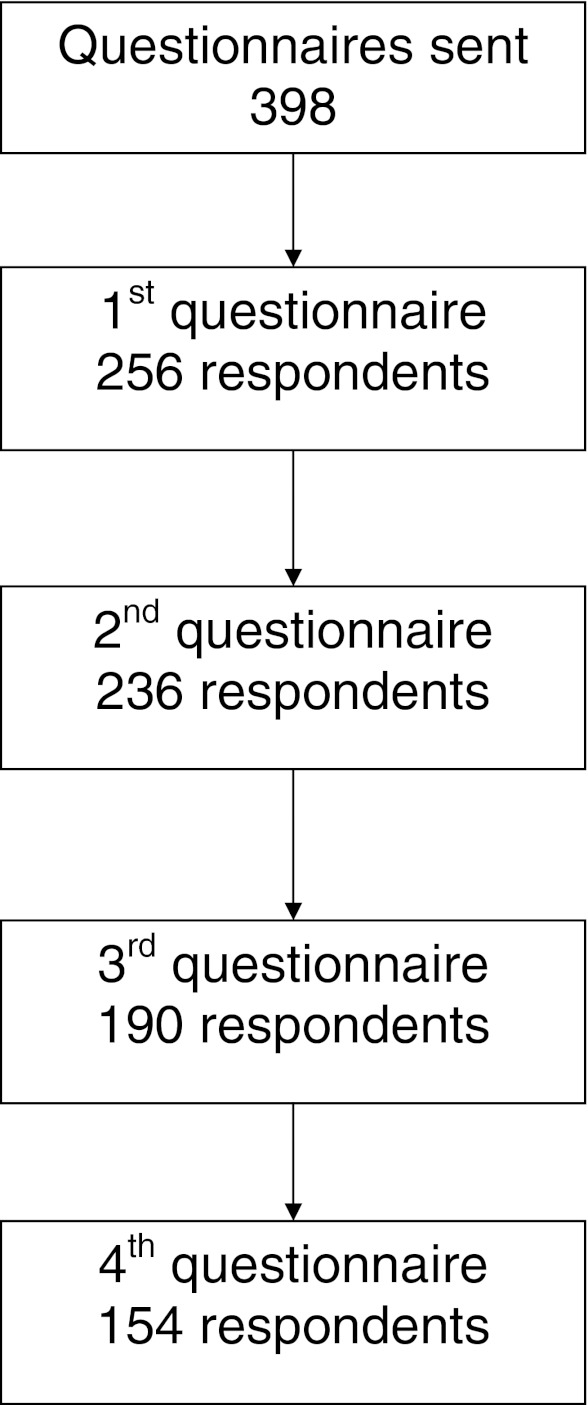
Response rate of the questionnaires

Of the 398 patients who registered for the study, 303 patients (76.1 %) filled in at least one questionnaire. Since patients were allowed to skip questionnaires, the number of the respondents to the first questionnaire is lower than the total number of respondents. For an overview of the response rate see Fig. 1. There were no statistically significant differences found regarding sex, age, and daily dosage between patients filling in a questionnaire compared with the patients who only registered for the study.

Two hundred and thirty-nine of the patients who filled in at least the first questionnaire reported an adverse drug reaction (78.9 %). In total, 152 different adverse drug reactions were reported. The reported adverse drug reactions, in absolute number as well as percentages, are presented in Table 1. Serious adverse drug reactions were reported by 4 patients (1.3 %). One was categorised as life- threatening, 2 required hospitalisation and 1 patient died. For an overview of these reactions see Table 2.

**Table 1:** The reported adverse drug reactions, in absolute numbers as well as percentages if *n* > 5

ADR	Number of patients	Percentage of patients
Nausea	79	26.1
Dry mouth	36	11.9
Dizziness	35	11.6
Hyperhydrosis	32	10.6
Headache	31	10.2
Somnolence	29	9.6
Constipation	29	9.6
Fatigue	29	9.6
Insomnia	26	8.6
Sleep disorder	24	7.9
Decreased appetite	19	6.3
Diarrhoea	15	5.0
Decreased libido	13	4.3
Micturition disorder	10	3.3
Malaise	10	3.3
Yawning	10	3.3
Anxiety	8	2.6
Weight increase	8	2.6
Restlessness	7	2.3
Erectile dysfunction	7	2.3
Myalgia	7	2.3
Vision blurred	7	2.3
Upper abdominal pain	7	2.3
Paraesthesia	6	2.0
Restless legs syndrome	6	2.0
Abnormal dreams	6	2.0
Tremor	6	2.0
Pollakiuria	5	1.7
Feeling abnormal	5	1.7
Tinnitus	5	1.7
Dysgeusia	5	1.7
Palpitations	5	1.7

**Table 2:** An overview of serious adverse drug reactions

Sex, age	Type of seriousness	Suspected adverse drug reaction	Concomitant medication	Time to onset, action with drug outcome	Comment
Female, 55	Hospitalisation	Suicide attempt, dry mouth, constipation	Zopiclone, propranolol, clorasepate	27 days for the suicide attempt, a few days for the dry mouth and constipation, the drug was withdrawn, patient is recovering	Patient used duloxetine for depression, treatment with ECT has been initiated
Female, 54	Hospitalisation	Constipation, headache, loss of appetite	Quetiapine, oxazepam	26 days for the loss of appetite, 4 days for the constipation, a few hours for the headache, the drug was withdrawn, the patient has recovered from the loss of appetite, but has not recovered from the two other events	Patient used duloxetine for depression, hospitalisation was because of drug use; however, it is not clear what the specific reason for the hospitalisation was
Female, 28	Life-threatening	Suicidal ideation, impulsive behaviour, feeling sad, restlessness, paranoid reaction, compulsions	Oxazepam	All reactions occurred in the first month, the drug dose was not changed. The outcome of the suicidal ideation and the restlessness is unknown, the patient has recovered from all other events.	Patient used duloxetine for depression
Male, 54	Death	Hyperhydrosis, dehydration, electrolyte disturbances, coma, death	None reported	One month, patient died	Patient used duloxetine for depression. The death was reported by the patient's partner

Of the 71 ADRs that were reported two or more times with the LIM system, 52 are explicitly mentioned in the EPAR for duloxetine.

### Signals

Events not labeled in the EPAR and events already labeled and for other reasons considered to be of interest (e.g., incidence differences) were analysed on a case by case basis.

#### Amenorrhoea

In the LIM cohort 2 cases of amenorrhea were reported. The first report concerns a woman aged 49 who experienced amenorrhea 20 days after the start of duloxetine for the treatment of neuropathic pain. The menstruation returned after withdrawal of duloxetine. Concomitant medication was several inhalation drugs (salbutamol/iptratropium, budesonide, formoterol), montelukast, esomeprazole, oxycodone and calcium carbonate/colecalciferol. The second report concerns a woman aged 45 who experienced amenorrhea just after the start of duloxetine for the treatment of depression. After missing two periods, the menstruation returned without change in duloxetine dose. No concomitant medication was reported.

#### Shock-like paraesthesia

In the EPAR the general term paraesthesia is mentioned as an ADR. In the LIM cohort two reports of electric shock sensation, a special form of paraesthesia, were received. The first report concerns a man aged 35 who experienced “small electric shocks” in the head 3 days after starting duloxetine for the treatment of depression. Upon reducing and eventually stopping the drug, the patient recovered. Concomitant medication consisted of lormetazepam and losartan/hydrochlorothiazide. The second report concerns a female aged 36 who experienced “a voltage in the brain” on the day of starting duloxetine treatment for depression. The patient recovered upon discontinuation of duloxetine. Comcomitant medication comprised oxazepam and ethinylestradiol/gestoden.

#### Micturition problems

The EPAR of duloxetine mentions urinary disorders as uncommon (frequency 0.1–1 %), except dysuria (frequency 1–10 %). In the LIM cohort urinary disorders were reported more frequently; a total of 17 patients (5.6 %) reported urinary disorders. Ten patients reported urinary hesitation, sometimes in combination with a decreased urine flow. Seven patients reported an increase in the micturition frequency. Of the 17 patients with urinary disorders, 11 were men and 6 were women. In 5 cases a positive dechallenge was reported; in another 5 cases the problems seem to disappear while continuing duloxetine treatment. In 4 cases the drug was continued and the patient did not recover. In 2 cases the duloxetine was withdrawn, but the patient had not (yet) recovered. In 1 case the outcome was not reported.

## Discussion

Web-based intensive monitoring gives an overview of the safety profile of duloxetine in daily practice as well as capturing the characteristics of its users.

### User characteristics

In the study the majority (69.1 %) of participants were female, which is consistent with the fact that both depression and neuropathic pain are more prevalent in women [16, 17]. The ages ranged from 14 to 82 years with 2 patients below 18 years of age. Duloxetine is registered for use in adults [1] and this shows that duloxetine, although it is a relatively new drug, is prescribed to younger patients off label. The majority of patients started with the 30-mg capsule and the average daily dosage was 49.1 mg, which is low compared with the recommended starting dosage of 60 mg once daily for the treatment of depression and diabetic peripheral neuropathic pain [1]. In this study duloxetine is used mostly as a treatment for depression; only 16.1 % of the patients used duloxetine for neuropathic pain. This is quite surprising since there were many treatment options for depression on the market at the time of the introduction of duloxetine, but few drugs registered for the treatment of neuropathic pain. However, many of the patients who received duloxetine stated that they had used other drugs for the same indication in the past, and SSRIs, together with TCAs and other antidepressant drugs were the most frequently mentioned, indicating that the patients who receive duloxetine did not respond to treatment with other antidepressant drugs. Another possibility is that the prevalence of depression is higher than the prevalence of neuropathic pain. It is surprising that almost 5 % stated that they used duloxetine for the treatment of fibromyalgia, even though this is not a registered indication in the European Union; however, in the USA duloxetine is indicated for the treatment of fibromyalgia [18]. Just as the intensive monitoring study of pregabalin, which is another drug indicated for the treatment of neuropathic pain, showed [12], it seems that duloxetine is prescribed to patients with fibromyalgia off label in the Netherlands.

### Adverse drug reactions

The ADRs most frequently reported in this study correspond to the most frequently reported ADRs in pre-registration trials, as well as in other trials [2–6, 19, 20]. The frequencies obtained with the LIM system are similar to those stated in the EPAR, except for a few cases. Of the possible ADRs reported via the web-based intensive monitoring system, three are worth additional attention.

Two reports of amenorrhea were reported. Even though the age of the patients (49 and 45 years old respectively) suggest that the amenorrhea could be due to the women entering the menopause, the absence of other symptoms relating to the menopause as well as the positive dechallenge in one case supports a causal relationship. Amenorrhea is not listed in the EPAR of duloxetine (the unspecified menopausal symptoms are), but can be explained from a mechanistic point of view as it is a clinical manifestation of hyperprolactinaemia that is mentioned in the EPAR and is caused by raised levels of serotonin, which is a modulator of prolactin secretion [21].

Shock-like paraesthesia consists of sensory perceptions of short electric low-voltage discharges, usually localised in the brain. In addition to these two reports, the Netherlands Pharmacovigilance Centre received three reports of shock-like paraesthesia through their spontaneous reporting system, strengthening this signal [22]. Shock-like paraesthesia has been described with the use of SSRIs [23, 24]. The symptoms usually occur during drug withdrawal, but have also been described with ongoing therapy. As it might not always be recognised as an ADR by patients and health care professionals, it is worth paying extra attention to it.

The EPAR for duloxetine mentions urinary disorders except dysuria as being uncommon (frequency 0.1–1 %). In the LIM cohort urinary disorders were reported more frequently; a total of 17 patients reported urinary disorders, mainly urinary hesitation and increased micturition frequency. It is notable that 11 of the 17 patients (64.7 %) with urinary problems were men, since only 30 % of the cohort are men. Only one of the men reported the use of drugs for treatment of benign prostate hypertrophy, which might be a confounding factor for the urinary disorders. The low frequency in the EPAR is surprising, especially for urinary hesitation, since duloxetine is registered under another brand name (Yentreve^®^), which is indicated for stress urinary incontinence [25].

### Strengths and weaknesses

Web-based intensive monitoring is an observational prospective cohort study mirroring the use and ADRs of duloxetine in daily practice compared with clinical studies, which have strict inclusion and exclusion criteria.

Eligible patients were identified in community pharmacies; however, not all patients eligible for inclusion participated in the study. Data on duloxetine dispensing during the inclusion period were provided by the Dutch Foundation for Pharmaceutical Statistics [26] and the LIM response rate was 3.5 % of all patients receiving a first prescription of duloxetine during the inclusion period. This might contribute to non-response bias. There is no information about the patients who did not participate; it is therefore not possible to know if the patients eventually participating in the study are representative of all patients using duloxetine. Non-response bias in a LIM study has been investigated and it showed that patients participating in LIM are in general younger and use a little less co-medication (0.8) than non-responders (Härmark et al., submitted for publication). However it cannot be assumed that younger patients experience fewer ADRs than older patients [27, 28].

In this study it was chosen to use the patient as a source of information. This has the advantage that adverse drug reactions are reported by the person who has actually experienced the reaction. Since patients do not have any “professional filter” in what they report, compared with health professionals, it enhances the chance of finding new ADRs that would not be considered ADRs, and therefore not reported by health professionals. For example, shock-like paraesthesias is an ADR that is primarily reported by patients compared with health professionals at the Netherlands Pharmacovigilance Centre Lareb. Since the patient is the source of information, it might be difficult to obtain information about fatal outcomes. In this study we received one report with a fatal outcome, and this was reported by the patient’s wife, showing that patient-based tools can also collect information about fatal outcomes.

It is surprising that almost 80 % of the patients who filled in a questionnaire reported an ADR. This is a rather high percentage and it is possible that patients who experienced an ADR were more inclined to fill in a questionnaire than those who did not experience ADRs, but analyses showed no difference in gender, age, and daily duloxetine dosage between the groups. Another reason for the high percentage might be channeling. Forty percent of the participants had in the past used one or more drugs for the same indication. It is not known if they switched because of a lack of efficacy or because of ADRs. If the latter were the reason for switching, it can be assumed that these patients might have an increased susceptibility to ADRs compared with others.

## Conclusion

This study indicates that the ADR profile of duloxetine as reported by patients over 6 months in daily practice is similar to the profile described in the EPAR of duloxetine [1]. Four patients (1.3 %) experienced a serious adverse drug reaction, of which one was fatal because of electrolyte disturbances. In addition, three signals of a possible new adverse drug reaction were identified; namely, amenorrhoea, shock-like paraesthesias, and urinary disorders and these need to be further evaluated in more detail. Web-based intensive monitoring has been shown to be a useful and efficient method of gaining insight into the behavior of new drugs in daily practice.
